# The Impact of Job-Demand-Control-Support on Leptin and Ghrelin as Biomarkers of Stress in Emergency Healthcare Workers

**DOI:** 10.3390/nu14235009

**Published:** 2022-11-25

**Authors:** Jean-Baptiste Bouillon-Minois, Justin Outrey, Bruno Pereira, Oluwaseun John Adeyemi, Vincent Sapin, Damien Bouvier, David Thivel, Sarah de Saint-Vincent, Ukadike Chris Ugbolue, Julien S. Baker, Reza Bagheri, Jeannot Schmidt, Marion Trousselard, Frédéric Dutheil

**Affiliations:** 1 Emergency Department, CHU Clermont-Ferrand, Université Clermont Auvergne, CNRS, LaPSCo, Physiological and Psychosocial Stress, F-63000 Clermont-Ferrand, France; 2 Emergency Department, CHU de Besançon, F-25000 Besançon, France; 3 Clinical Research and Innovation Direction, CHU Clermont-Ferrand, F-63000 Clermont-Ferrand, France; 4 Ronald O. Perelman Department of Emergency Medicine, NYU School of Medicine, New York University Langone Health, New York, NY 10016, USA; 5 Centre de Biologie, CHU Clermont-Ferrand, F-63003 Clermont-Ferrand, France; 6 Research Center in Human Nutrition, Laboratory AME2P, Université Clermont Auvergne, F-63120 Aubière, France; 7 Occupational and Environmental Medicine, Université Clermont Auvergne, F-63000 Clermont-Ferrand, France; 8 School of Health and Life Sciences, Institute for Clinical Exercise & Health Science, University of the West of Scotland, Glasgow G1 1XW, UK; 9 Centre for Health and Exercise Science Research, Department of Sport, Physical Education and Health, Hong Kong Baptist University, Kowloon Tong 999077, Hong Kong; 10 Department of Exercise Physiology, University of Isfahan, Isfahan 81746-73441, Iran; 11 Neurophysiology of Stress, French Armed Forces Biomedical Research Institute, IRBA, F-91223 Brétigny-sur-Orge, France; 12 CHU Clermont-Ferrand, Université Clermont Auvergne, CNRS, LaPSCo, Physiological and Psychosocial Stress, Occupational and Environmental Medicine, WittyFit, F-63000 Clermont-Ferrand, France

**Keywords:** nutrients, work, well-being, quality of life, leptin, ghrelin

## Abstract

Despite the available literature on the consequences of night shiftwork on stress and food intake, its impact on leptin and ghrelin has never been studied. We previously demonstrated that leptin and ghrelin were biomarkers related to stress, and acute stress-induced a decrease in leptin levels and an increase in ghrelin levels. We performed a prospective observational study to assess the influence of night work, nutrition, and stress on the levels of ghrelin and leptin among emergency healthcare workers (HCWs). We took salivary samples at the beginning of a day shift and/or at the end of a night shift. We also monitored stress using the job demand-control-support model of Karasek. We recorded 24-h food intake during the day shift and the consecutive night shift and during night work and the day before. We included 161 emergency HCWs. Emergency HCWs had a tendency for decreased levels of leptin following the night shift compared to before the dayshift (*p* = 0.067). Furthermore, the main factors explaining the decrease in leptin levels were an increase in job-demand (coefficient −54.1, 95 CI −99.0 to −0.92) and a decrease in job control (−24.9, −49.5 to −0.29). Despite no significant changes in ghrelin levels between shifts, social support was the main factor explaining the increase in ghrelin (6.12, 0.74 to 11.5). Food intake (kcal) also had a negative impact on leptin levels, in addition to age. Ghrelin levels also decreased with body mass index, while age had the opposite effect. In conclusion, we confirmed that ghrelin and leptin as biomarkers of stress were directly linked to the job demand-control-support model of Karasek, when the main cofounders were considered.

## 1. Introduction

Stress at work is a major public health concern. This is especially true among healthcare workers (HCWs) and even more concerning in the Emergency Departments (ED) due to shifting work, fatigue and lack of sleep [1], poor food intake [2], and life-threatening emergencies in the context of overcrowding and job demands [3,4]. Currently, the best scale to assess stress levels at work is the job-demand-control-support model (JDCS), a self-reported psychological questionnaire created and validated by Karasek in 1981 [5,6]. Night work, defined as “all work which is performed during a period of not less than seven consecutive hours, including the interval from midnight to 5 a.m.” [7], has many health consequences. These include disturbance of circadian rhythm [8], obesity, and cardiometabolic disorders [9,10,11] which are putative consequences of eating disorders [12] and stress [13]. Eating behavior can be influenced by sociodemographic criteria such as age and gender [14] and by occupational characteristics such as experience [15] and workload [16,17]. Night work also induces a conflict between socially determined diurnal mealtimes, eating habits, the biological rhythm of hunger, satiety, and metabolism [18,19]. The main hormones that control the physiology of appetite are ghrelin and leptin. Ghrelin leads the orexigenic role [20]. Increased ghrelin levels influence meal initiation and food-seeking behavior [21]. In response to a meal, levels vary quickly and intensely due to its short half-life [22,23]. Leptin, a peptide mainly produced in white adipose tissue, is commonly known as a satiety hormone, with low levels observed during meal initiation and high levels after the meal [24]. Its baseline blood levels are influenced by body mass index (BMI), mainly due to the white adipose tissue component [25]. Although the classical hormones to assess stress are cortisol, epinephrine, interleukin and melatonin, both ghrelin and leptin are influenced by stress and can be considered biomarkers of stress [26,27,28,29,30,31]. Indeed, we previously performed a meta-analysis on the impact of both physical and psychological stress on ghrelin and leptin levels, measured using both blood or saliva. We found that ghrelin has a short-term increase following acute stress with a higher and prolonged response among obese individuals [27]. Regarding leptin, we found a decrease following acute stress with a higher variation of leptin levels after stress among normal-weight individuals and women [26]. Emergency HCWs are a perfect example for studying the impact of night shift and stress on biomarkers of nutrition [32]. Indeed, HCWs work under stressful conditions such as overcrowding—lack of beds in hospitals [3], life-threatening emergencies [33], the wait for possible disasters [34] all having consequences on the biomarkers of stress [31,35]. Furthermore, the night shift has a bad influence on water consumption and food intake among emergency HCWs, in both quantity and quality [2]. To date, no studies have assessed the influence of night work and stress on the levels of nutrition biomarkers among emergency HCWs, nor in relation to their occupational characteristics.

Therefore, we performed this study to assess the influence of night work, stress, and sociodemographic characteristics on ghrelin and leptin levels among emergency HCWs.

## 2. Materials and Methods

### 2.1. Study Design

**Figure 1:**
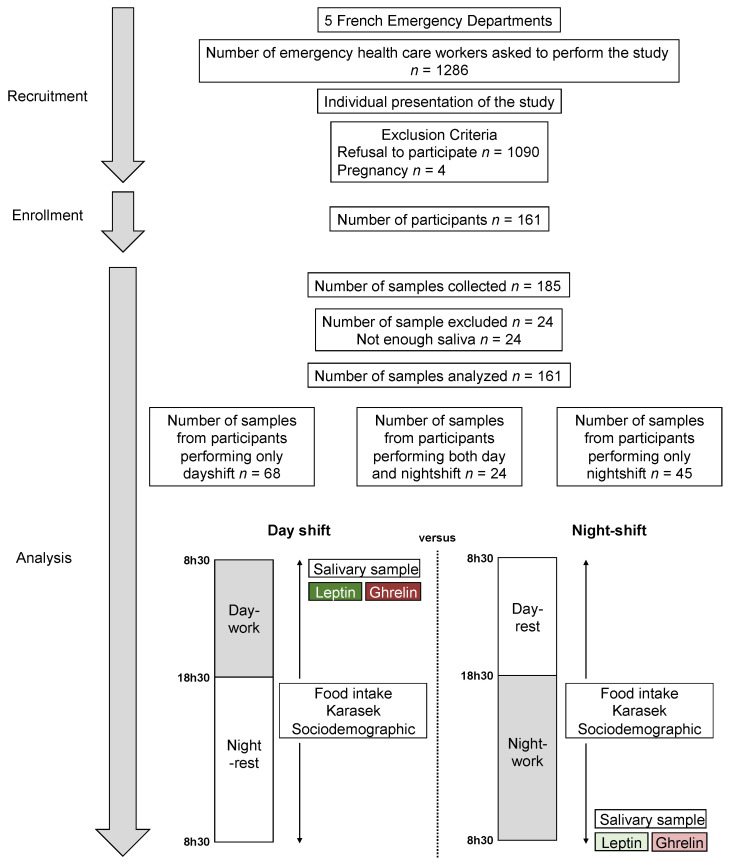
Study design. Among five French Emergency Departments, we were able to recruit 161 emergency healthcare workers for a total of 185 salivary samples. Twenty-four were excluded because of a low quantity of saliva. Ghrelin and leptin levels were assessed from 161 samples.

We performed a prospective nationwide observational study in five French hospitals—two university hospitals and three non-university hospitals. The main inclusion criterion was to work as an emergency HCW. Exclusion criteria were refusal to participate and pregnancy. This study was part of the SEEK protocol [2,32,34]. We obtained ethical approval from the French Ethics Committee South-East I with reference DC-2014-2151, and the protocol was registered on ClinicalTrials.gov, number NCT02401607. The study was performed during two shifts (day shift and night shift). During the day shift, participants started to work between 7:30 and 8:30 a.m. and finished between 6:30 and 7:30 p.m. For nightshift, they began between 6:30 and 7:30 p.m. and finished between 7:30 and 8:30 a.m. the following day. All participants volunteered and signed consent forms before answering questionnaires and providing samples. They performed the study for one dayshift and/or one nightshift. Salivary sampling was collected at the beginning of a dayshift and at the end of a nightshift, i.e., between 7:00 a.m. and 8:30 a.m. for both conditions. Furthermore, food intake was monitored twice over 24 consecutive hours: (1) during a day shift (from 8:30 a.m. to 6:30 p.m.) + the night after (no work, 6:30 p.m. to 8.30 a.m.), and (2) during a rest day before (from 8:30 a.m. to 6:30 p.m.) and a night shift (6:30 p.m. to 8:30 a.m.) [2]. Participants also had to complete a questionnaire collecting their level of stress using the JDCS model of Karasek [6] and sociodemographic data (Figure 1).

### 2.2. Outcomes

Ghrelin and leptin levels were measured from saliva samples. We used saliva preferenttialy because its collection is non-invasive and results are acceptable [36]. Saliva samples were stored at −80 °C at the Institute of Occupational Medicine of Clermont-Ferrand. Total ghrelin was assessed by ELISA using commercial kits (CEA991Hu Clound Clone Corp^®^ (CLOUD-CLONE CORP. (CCC, Los Angeles, CA, USA)) with a detection range from 12.35 to 1000 pg/mL, a sensitivity level of <4.87pg/mL, an intra-assay coefficient < 10% and an inter-assay coefficient < 12%. Leptin was assessed using ALPCO, Salem, NH, USA/22-LEPHUU-e01/kit ultrasensitive with a range from 0.05 to 5 ng/mL, a sensitivity level at 0.01 ng/mL, an intra-assay coefficient at 7.2 % and an inter-assay coefficient at 4.35%. Karasek’s job-content questionnaire (JCQ) is composed of three dimensions: psychological demands, decision latitude, and social support. Participants were assessed by the 26 items of the JCQ (nine for both decision latitude and psychological demand and eight for social support). Each participant was asked to answer using a 4-level Likert-type scale for each item, ranging from 1 (strongly disagree) to 4 (strongly agree). Decision latitude was calculated using the following formula: 4 × Q4 + 4 × (5 − Q6) + 4 × Q8 + 2 × (5 − Q2) + 2 × Q5 + 2 × Q7 +2 × Q1 + 2 × Q3 + 2 × Q9. A score less than 71 reflects low decision latitude. Psychological demand was calculated with the following formula: Q10 + Q11 + Q12 + (5 − Q13) + Q14 + Q15 + Q16 + Q17 + Q18. A score below 20 reflected a low psychological demand. Social support was calculated with the following formula: Q19 + Q20 + Q21 + Q22 + Q23 + Q24 + Q25 + Q26. A score below 24 reflected low social support [6]. Participants were also asked to complete a 24-h dietary recall that was explained and detailed to them by a member of the investigation team. The participants were asked to indicate as precisely as possible all the details regarding the food ingested during each meal and in-between meals. The diaries were reviewed with the participants during a dedicated interview. We next used Nutrilog^®^ (Nutrilog, version 3.2), a diet and nutrition software for health care professionals, to translate participants’ answers on nutrient intake using a nutrition table Ciqual based on this software. We calculated total energy intake (kcal), carbohydrates, lipids, proteins (grams and % energy intake), and water consumption in milliliters (mL) [2]. Participants also had to complete a questionnaire about their sociodemographics (age, gender, marital status, kids at home, tobacco) and work situation (occupation, seniority).

### 2.3. Statistics

Continuous variables were expressed as mean and standard deviation (SD), or median [interquartile range] (IQR), and categorical variables were expressed as numbers (percentage—%). The assumption of Gaussian distribution was analyzed by the Shapiro-Wilk test. As data could be repeated for a part of participants’ habits (measures for nightshift and dayshift), the linear mixed method was performed (i) to compare values between nightshift and dayshift (i.e., to assess the influence of night work on ghrelin and leptin levels) and then (ii) to assess the influence on ghrelin and leptin levels on stress (using job demand, job control, and social support), nutrition (food intake in kcal, carbohydrates, lipids and proteins in grams), water consumption and sociodemographic data (job, age, BMI). Time (nightshift versus dayshift) was considered as a fixed effect, whereas participants were considered as random effects in order to model between and within-subject variability. The normality of residuals was analyzed with the Shapiro-Wilk test and a visual plot. Accordingly, ghrelin and leptin levels were log-transformed. The results were expressed as coefficient regressions and 95 confidence intervals (95 CI). To increase readability, results were expressed using the following formulas: 1000 × log(leptin) or 100 × log(ghrelin). Statistical analyses were performed with Stata software (v17, College Station, TX, USA). Significance was set at the *p* < 0.05 level. Sensitivity analysis was conducted among emergency HCWs that engaged in the study on both the dayshift and nightshift. Both were compared for their main characteristics to evaluate the sample representativeness.

## 3. Results

### 3.1. Characteristics of the Population

We enrolled 161 emergency HCWs from five hospitals and obtained a total of 185 samples of saliva. Twenty-four samples were excluded due to the insufficiency of saliva. In total, we analyzed 161 saliva samples. Twenty-four (14.9%) performed the study twice (one during the day shift and one during the night shift) (Figure 1). The representativeness of the emergency HCWs was examined. We did not find any differences between the HCWs that performed the study twice versus those who performed only one shift. Fifty-seven HCWs were physicians, 79 were nurses, and 24 had other occupations (administrative, maid). Participants were 37.4 ± 10.4 years old, mainly female (57.5%, sex ratio = 1.37), with a mean BMI of 23.2 ± 4.4 kg/m^2^. Fifty-three (32.9%) were single, and 77 (58.3%) had no children. Median seniority as a HCWs was 6 [IQR 13] years, 3 [9] years in the ED (Table 1).

### 3.2. Assessment of Ghrelin and Leptin Levels

**Figure 2:**
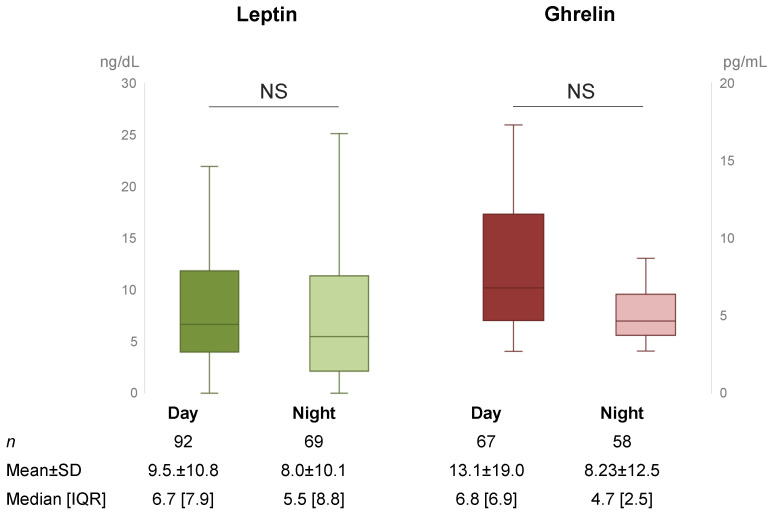
Median [interquartile range] salivary levels of leptin and ghrelin before the beginning of a day shift and at the end of a night shift. The results of leptin are expressed in nanograms per deciliter. Results of ghrelin are expressed in picogram per milliliter. Ng = nanogram, pg = picogram, dL = deciliter, mL = milliliter, NS = not significant, n = number of samples studied, SD = standard deviation, IQR = interquartile range.

We did not find any statistically significant change on the level of both ghrelin and leptin after the end of the night shift compared to the beginning of the day shift (Figure 2).

### 3.3. Impact of Stress, Night Shift, Sociodemographic, and Nutrition on Leptin and Ghrelin Levels

**Figure 3:**
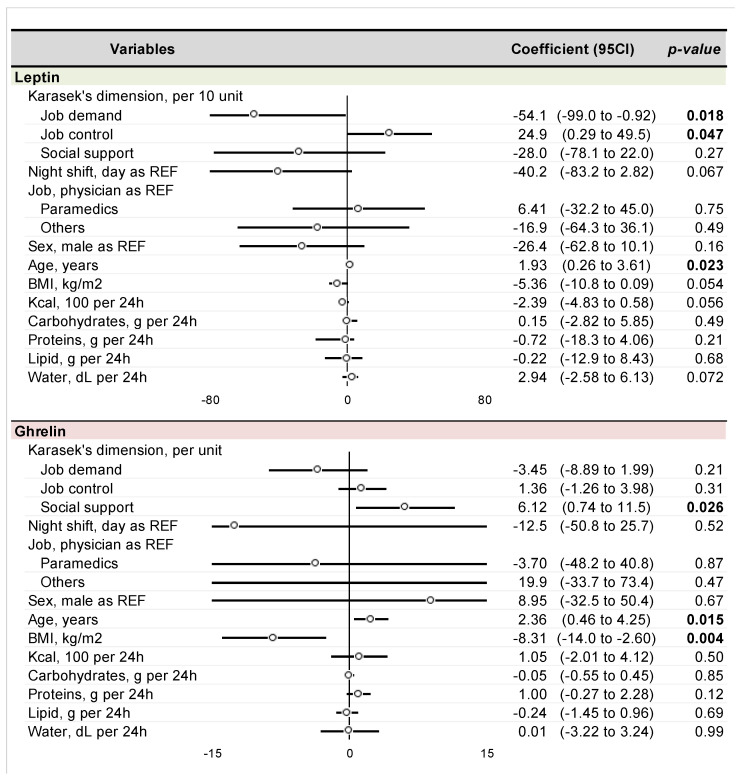
Impact of stress assessed using Karasek jobdemand-control-suport, night, sociodemographic status and nutrition on leptin and ghrelin salivary levels using a linear mixed model. Job demand refers to the psychological needs imposed by daily working activities, job control refers to the latitude of decision and is composed of two components: skill discretion and decision authority. Social support represents the support from colleagues and/or from the hierarchy. Results were expressed using coefficient regression (95% confidence interval). Results were considered significant for *p*-value < 0.05 and 95 CI not containing 0. BMI = body mass index, kg = kilogram, m^2^ = square meter, g = gram, dL = deciliter.

Regarding leptin levels (expressed as 1000 × log leptin)—we found a negative impact of job demand on leptin levels (Coefficient −54.1 per 10-unit; 95 CI −99.0 to −0.92) while job control increased leptin levels (24.9 per 10-unit; 0.29 to 49.5). Age increases parallelly with leptin level (1.93 per year; 0.26 to 3.61). We also found a negative tendency for night shift (−40.2; −83.2 to 2.82), BMI (−5.36 per kg/m^2^; −10.8 to 0.09) and food intake (−2.39 per 100 kcal/24 h; −4.83 to 0.58) while water consumption tended to increase leptin levels (2.94 per dL/24 h; −2.58 to 6.13). Regarding ghrelin (expressed as 100 × log ghrelin), we found a positive impact of social support (6.12 per unit; 0.74 to 11.5), and age (2.34 per year; 0.46 to 4.25) and a negative impact for BMI (−8.31 per kg/m^2^; −14.0 to −2.60) (Figure 3).

## 4. Discussion

We demonstrated that both stress, nightshift, and food intake have an impact on nutrition biomarkers represented by ghrelin and leptin.

### 4.1. The Impact of Night Shift and Stress

We found a lower level of both ghrelin and leptin after the night shift compared to before the beginning of the day shift. Considering that these hormones act inversely, this result needs to be discussed. Ghrelin is considered the main orexigenic hormone. A high level of ghrelin promotes food intake, while a low level is observed at the end of a meal. Literature on the impact of night work on ghrelin levels is not strong and is controversial. Indeed, one study did not find any difference in 24 h ghrelin levels recorded between day and night shifts [37], while another study found a lower ghrelin level among night shift workers compared to dayshift workers [38]. However, all studies observed that ghrelin levels are unstable (in a range of minutes) to be considered using mean levels. Conversely, leptin is the main satiety hormone, with low levels observed during meal initiation and high levels after the meal. However, the impact of night work on leptin levels seems to be negative. Indeed, it seems that average 24 h leptin levels decreased up to 40% during the night shift compared to baseline in two studies that experimentally created night and day shifts [37,39]. Furthermore, this impact seems to increase for participants that have a non-synchronized schedule [40]. However, a further study noted different results, i.e., an increase in leptin levels among hospital nurses among night workers that worked exclusively during the night for at least one year [41]. Regarding the impact of stress on ghrelin, we did not find any significant impact on job demand and job control. However, it seems that social support increases ghrelin levels (coeff 6.12; 95 CI 0.74 to 11.5). Regarding leptin, job demand decreased leptin levels while job control increased levels. This confirms the influence of stress on leptin levels and, more widely, the impact of stress on biomarker responses. We previously published that both ghrelin and leptin, measured using both blood or saliva, are influenced by stress. More precisely, ghrelin increases very quickly once the stress (physical, psychological of both) is induced—within less than five minutes—and returns to baseline levels within 45 min with a higher and prolonged response among obese individuals. Leptin decreases more slowly, with a significant decrease after 60 min, with a higher variation of leptin levels after stress among normal-weight individuals and women [26,27].

### 4.2. Impact of Food Intake and Sociodemographic Status

We found a negative impact of kcal per day intake on leptin levels with no impact on ghrelin levels. This result is not logical when initially considered. Indeed, leptin is a satiety hormone, so we would expect that greater amounts of food would induce higher levels of leptin. Furthermore, we also found a negative impact of BMI (coef −0.005; 95 CI −0.011 to 0.0001). Indeed, overweight individuals have a higher circulating leptin level and leptin resistance due to the availability of highly palatable food that increases triglyceride production and, therefore, white adipose tissue production of leptin [42,43]. A possible explanation for this could be the negative impact of sleep deprivation. Indeed, several studies found lower leptin levels among healthy volunteers with acute sleep deprivation or chronic partial sleep deprivation [44,45,46]. Very interestingly, we did not find any impact of qualitative nutrients, i.e., carbohydrates, lipids, or proteins, on biomarker levels. Furthermore, water consumption seems to increase leptin levels, although we did not find an explanation for why in the literature. A possible answer is that participants declared drinking soft drinks, not water, a high-concentrated fructose drink, is linked to obesity [47]. Lastly, we found a higher level of leptin among older participants, which is consistent with the literature [48]. Regarding ghrelin, we did not find significant covariates. This could be explained by fluctuations in homeostasis. We only found a negative correlation between BMI and ghrelin level, which is consistent with the literature [49]. However, we found a positive impact of age on ghrelin levels, which is controversial in the literature [50,51,52]. No other data was significant regarding food intake, water consumption, stress, or quality of food.

### 4.3. Limitations

Our study has some limitations, none of which affects our results. First, we performed only one measurement, but both ghrelin and leptin have a circadian rhythm [53]. This rhythm can be disturbed by night-shift. Furthermore, the two sessions have different periods. The day-shift is four-hours shorter than the night-shift. However, every sample was collected and studied under the same conditions. Second, total ghrelin was assayed rather than its active, acylated form. Nevertheless, most of the physiology of ghrelin has been defined based on the assay of total ghrelin. Thirdly, even if we tried to monitor the time of breakfast, data were too imprecise to be used. Indeed, HCWs eat their breakfast before work during the day shift, but it is not consistent with those working on the night shift. Considering that ghrelin levels decrease quickly and intensely after the beginning of food intake and increase slowly until lunch, the putative results could be more significant. Some devices are now able to continuously monitor certain biomarkers. Advances in technology should soon facilitate the circadian rhythm of leptin and ghrelin in emergency HCWs, making it feasible to study the underlying mechanisms of stress regulation.

## 5. Conclusions

In conclusion, we did not find any difference between night and day regarding ghrelin and leptin levels. However, we found that ghrelin and leptin can be considered as biomarkers of stress directly linked to the job demand-control-support model of Karasek, in a study that considered the main cofounders. Understanding mechanisms of stress in a comprehensive study may help to build efficient health promotion and/or preventive strategies.

## Figures and Tables

**Table 1:** Sociodemographic. SD = standard deviation, BMI = body mass index, kg = kilograms, kg/m^2^ = kilogram per square meter, IQR = interquartile range, *n* = number, % = percentage. Results are expressed as mean ± standard deviation or median [interquartile range] or number (percentage).

	Total *n* = 161	Physician *n* = 57	Paramedics *n* = 79	Other *n* = 24
Age, years, mean ± SD	37.4 ± 10.4	35.7 ± 10.4	39.5 ± 10.2	34.9 ± 10.3
Sex, *n* (%) male	68 (42.5)	29 (50.9)	28 (35.4)	11 (45.8)
BMI, kg/m^2^, mean ± SD	23.2 ± 4.4	22.9 ± 4.3	23.2 ± 3.6	23.7 ± 4.4
Underweight, *n* (%)	10 (6.2)	3 (5.3)	6 (7.6)	1 (4.2)
Normal weight, *n* (%)	107 (66.5)	39 (68.4)	53 (67.1)	15 (62.5)
Overweight, *n* (%)	36 (22.4)	14 (24.6)	17 (21.5)	5 (20.8)
Obesity class 1, *n* (%)	4 (2.5)	0	1 (1.3)	3 (12.5)
Obesity class 2, *n* (%)	1 (0.6)	0	1 (1.3)	0
Obesity class 3, *n* (%)	3 (1.9)	1 (1.8)	1 (1.3)	0
Seniority, years, median [IQR]				
In the job	6 [13]	4 [8.5]	11 [13]	4.5 [8.5]
In the department	3 [9]	2 [4.5]	5 [9]	1 [6]
Physical activity, h/week, median [IQR]	2 [4]	2 [5]	2 [4]	2 [3]
Tea/coffee, cup/day, median [IQR]	3 [3]	4 [2]	6 [12]	5 [3.5]
Smoker, *n* (%)	57 (35.6)	17 (29.8)	31 (39.7)	9 (37.5)
Cig/day for smokers, median [IQR]	6 [9]	8 [5]	6 [12]	5 [3.5]
Family situation				
Married-engaged, *n* (%)	108 (67.1)	35 (61.4)	63 (79.7)	9 (37.5)
Single-divorced, *n* (%)	53 (32.9)	22 (38.6)	16 (20.2)	15 (62.5)
Kids at home, *n* (%)	55 (41.7)	16 (33.3)	35 (53.0)	4 (22.2)

## Data Availability

Data are available by request to the corresponding author.
